# Answer to letter: dissociating advances in orthopaedic trauma management from the climbing patient expectations. “Good vs. good”—setting reasonable goals for patients’ satisfaction

**DOI:** 10.1007/s00068-021-01755-4

**Published:** 2021-07-29

**Authors:** Lena Keppler, Alexander Martin Keppler, Christoph Ihle, Philipp MInzlaff, Julian Fürmetz, Markus Beck, Tim Saier

**Affiliations:** 1 Department of Trauma Surgery, BG Trauma Center Murnau, Murnau, Germany; 2 Department of Orthopaedics and Trauma Surgery, University Hospital, LMU Munich , Munich, Germany; 3 Department of Traumatology and Reconstructive Surgery, BG Trauma Center Tuebingen, Tuebingen, Germany; 4 Department of Trauma and Orthopedic Surgery, Hospital Agatharied, Hausham, Germany; 5 Department of Orthopedic Surgery, Klinikum Rechts der Isar, Munich, Germany

To the Editor,

We thank Lari et al. for their letter to the Editor.

In their letter, the authors accurately describe the challenges facing modern surgical orthopaedics and traumatology.

The reduction of major trauma mortality in the last decades is a great success of our field [1]. By constantly improving surgical techniques, implants, and individual post-treatment concepts, it is possible to provide not only severe monotrauma but also polytraumatized patients with better care. As a result, there is now not only a higher chance of survival after severe injuries, but these patients are also potentially able and willing to return to work or to engage in sports.

However, due to the fortunately increasing proportion of high-quality medical care, the demands of patients across all age groups are also becoming even higher.

For patients, it is not the classic, often abstract, objective parameters such as adequate fracture healing or reconstruction of the joint surface that play a central role in their healing process, but much more “real” and “everyday-relevant” criteria and goals such as the ability to kneel, climb stairs or the ability to return to sports or work.

As physicians, we have to adequately meet these expectations already preoperatively. Here, not only patient-physician communication plays an important role, but also the patient's origin and culture [2].

For us as treating physicians, the guiding principle is quality before quantity. The quality of our work refers primarily to objective parameters. We do not only strive for guideline-based therapy, but at the same time, we want to provide an individual, tailor-made treatment for each patient. To achieve this, we nowadays have to take into account much more the subjective parameters and expectations of the individual patient. After all, it is precisely these subjective expectations that are decisive for satisfaction with the treatment outcome. Their evaluation appears to be increasingly important against this background [3].

The outcome can already be measured very well in variables such as mobility or freedom from pain, both objectively and subjectively. By preoperatively inquiring about individual expectations, we as physicians can adapt our therapy even better, and in many cases take away unrealistic expectations, thus informing patients even better about the likelihood of success of an intervention.

Especially in our field, where the health status of our patients changes abruptly in a short period of time, an intensive examination of the respective expectations of the patients must take place. In our opinion, this is one of the cornerstones for successful treatment.

To paraphrase Alexander Pope: “Blessed is he who expects reasonable goals, for he shall never be disappointed”.
